# Impact of oral capsule of *Peganum harmala* on alleviating urinary symptoms in men with benign prostatic hyperplasia; a randomized clinical trial

**DOI:** 10.15171/jrip.2017.25

**Published:** 2016-12-18

**Authors:** Majid Shirani-Boroujeni, Saeed Heidari-Soureshjani, Zahra Keivani Hafshejani

**Affiliations:** ^1^Department of Surgery, Shahrekord University of Medical Sciences, Shahrekord, Iran; ^2^MSc, Research and Technology Deputy, Shahrekord University of Medical Sciences, Shahrekord, Iran; ^3^Department of Nursing, Shahrekord University of Medical Sciences, Shahrekord, Iran

**Keywords:** Lower urinary tract, Tamsulosin, Benign prostatic hyperplasia, *Peganum harmala*

## Abstract

**Introduction:** Benign prostatic hyperplasia (BPH) is considered as a major cause of lower urinary tract symptoms (LUTS) in older men and its most common sign is nocturia.

**Objectives:** This study aimed to determine the effect of the seeds of Peganum harmala compared with tamsulosin on alleviating urinary symptoms in patients with BPH.

**Patients and Methods:** In this single blind clinical trial study, 90 patients diagnosed with BPH and LUTS, based on international prostate standard survey (IPSS) were divided into three groups. The first group was received oral capsule of P. harmala, the second group was administered tamsulosin with oral P. harmala seed and the third group was received tamsulosin drug and they were evaluated after 4 weeks.

**Results:** The results showed that the difference between mean scores of IPSS was significant after the intervention (*P*=0.001). Besides, the mean of IPSS in the three groups was significantly different (P=0.001) (the first group 41.9±5.3, the second group 21.0±4.4 ,the third group 16.5±3.7 respectively). However, after the intervention, patients in the second group had the lowest average on most indicators of IPSS but the difference was only significant about urinary frequency, nocturia and intermittency(*P*<0.05).

**Conclusion:** Application of Peganum harmala seed can be useful in reducing urinary symptoms in patients with BPH.

**Implication for health policy/practice/research/medical education:**

Peganum harmala can be administered as a herbal remedy for improving urinary symptoms of benign prostate enlargement.

## Introduction

Benign prostatic hyperplasia (BPH) is occurred due to the proliferation of irregular connective tissue, glandular epithelium and smooth muscle in the prostate transition zone (1).

The disease involves tens of millions of elderly men in the world and as growing public health problem causes urinary retention (bladder outlet obstruction) (2).

Clinically, the disorder is diagnosed as lower urinary tract symptoms (LUTS) (3). Hence, the development of urinary tract symptoms is considered as BPH progression causing problems such as loss of quality of life of patients with acute urinary retention, recurrent infections of the urinary tract, urinary incontinence, calcium deposits in the bladder, kidney obstructive failure, BPH-related surgery (4) and probably threaten the patients’ life (5).

The treatment recommended preventing BPH progression was consisted of the combination of 5α-reductase inhibitors and α blocker (4). Tamsulosin is of the third generation of alpha-blockers and is regarded as one of the most frequent drugs used for BPH. Its long-term consumption is followed by complications such as orthostatic hypotension, dizziness, headache, asthenia, runny nose and ejaculation problems as well (4,6).

In recent years, the use of herbal medicines and natural products is increasing because of fewer side effects (7,8). On the other hand, effect of various herbal medicines on BPH and its associated complications has been reported in various studies (9-11).

*Peganum harmala* L. which belongs to *Zygophyllaceae* family, has long been focused in Iranian traditional medicine. *P. harmala* is a wild and perennial plant grown in a semi-arid, steppe regions and sandy soils in the Eastern Mediterranean region (12-14).

The seeds of this plant are rich in carbohydrates, lipids, proteins, minerals, alkaloids and amino acids. Active ingredients of this plant include mainly beta-carboline and quinazoline alkaloids that were particularly accumulated in its seeds and roots (14).

The properties of this plant were consisted of positive effects on cardiovascular disease, nervous system, gastrointestinal complications, bone, diabetes, immune system disorders, antimicrobial activity inducing abortion, anti-inflammatory, anti-cancer and anti-proliferative activities (14). However, it should be noted that excessive consumption of this herb and its illegal prescription can also cause various complications and toxicity in consumers (11).

Although diagnostic methods and new treatments for BPH management have been developed (15), there is no effective treatment with few side effects for this disorder.

## Objectives

Given the importance of this plant in herbal medicine and limitations of clinical studies, this study was conducted to determine the effect of edible seeds of *P. harmala* on nocturia in patients with benign prostatic hyperplasia referred to urology clinic affiliated with Ayatollah-Kashani hospital in Shahrekord, Iran.

## Patients and Methods

### Subjects

This double-blind clinical trial study was carried out on 90 patients diagnosed with benign prostatic hyperplasia based on international prostate standard survey (IPSS).

Besides, criteria such as the size of the prostate, prostate-specific antigen (PSA), history of previous disease, history of previous surgery were considered. Inclusion criteria were patients with benign prostatic hyperplasia. Exclusion criteria were patients who were candidate of surgery, obvious anatomic or functional anomaly in the urinary tract, thyroid disorder, prostate cancer, patients with mental disorders, active or chronic infections and sensitivity to the drug.

Patients were divided into three groups. The sampling method was random allocation.

The first group (32 patients) treated with oral capsules of *P. harmala* seed (containing one gram of *Pe. harmala*), the second group (29 patients) was received conventional therapy of alpha blockers (tamsulosin) in combination with oral administration of *P. harmala* and the third group (29 patients) were prescribed conventional therapy of alpha blockers (tamsulosin) alone. Therapeutic dose used in this study was 1 g. However, due to the hepatotoxicity effect of *P. harmala*, hepatic factors such as aspartate aminotransferase (AST), alanine aminotransferase (ALT), and alkaline phosphatase (ALP) in all patients were checked and after 4 weeks were examined.

### Ethical issues

The research followed the tenets of the Declaration of Helsinki; informed consent was obtained, and the research was approved by the ethics committee of Shahrekord University of Medical Sciences. In this study, the full description of the processes and the importance of the study were explained to the participants who had volunteered and were selected (IRCT code# IRCT2014091419151N1, and ethical approval code# 92-5-4).

### Statistical analysis

The data were analyzed by SPSS 22 using descriptive tests, Kruskal-Wallis, Wilcoxon and Mann-Whitney U tests. A *P* value less than 0.05 was considered as statistically significant.

## Results

In this study, 90 patients with benign prostatic hypertrophy were divided into three groups. Groups were not significantly different regarding to mean age, prostate size by ultrasound and PSA levels (*P* > 0.05).

Of the 90 patients, in *P. harmala* group 24 patients (75%), in tamsulosin and *P. harmala* group 24 (82.8 %) and in tamsulosin group 22 patients (75.9 %) had no previous medical history. The history of surgery was 27 (84.4%) in the* P. harmala* group, 26 (89.7%) in tamsulosin and *P. harmala* group. Twenty-six (89.7%) in tamsulosin group had no history of previous surgery.

The average size of the prostate in *P. harmala* group*, P. harmala* and tamsulosin group, and tamsulosin group was 25.81 ± 14.8 cm^3^, 25.71 ± 11.3 cm^3^, 75.03 ± 13.65cm^3^, respectively. The serum level of PSA in *P. harmala* group*, P. harmala* and tamsulosin group, and tamsulosin group was 1.37 ± 0.98 ng/mL, 1.64 ± 0.70 ng/mL, and 1.18 ± 0.77 ng/mL respectively.

Scores distribution in the scoring questionnaire was not normal. Thus, to compare the scores of three groups before and after the intervention, the Kruskal-Wallis test was used. Before the intervention, there was no significant difference in the average of the three groups (*P* = 0.347), which indicated that the groups were matched. However, significant differences in mean scores after the intervention was detected (*P* = 0.001).

The lowest score of the questionnaire after the intervention in tamsulosin and *P. harmala* groups was 12.0 ± 4.4 and most score was related to tamsulosin group with 16.5 ± 3.7.

Besides, it was shown that *P. harmala* and tamsulosin group with an IPSS average of 12.0 ± 4.4, *P. harmala* with an IPSS average 14.9 ± 5.3 and tamsulosin group with an IPSS average of 16.5 ± 3.7 were significantly different; however, *P. harmala* and tamsulosin group and tamsulosin group were not significantly different. In addition, it was indicated that in three groups before and after the intervention, mean scores were significantly different (*P* < 0.001; Table 1).

**Table 1:** Comparison of IPSS before and after intervention in all three groups based on the total survey score

	**Tamsulosin group**	***Peganum*** ***harmala*** ****** **and Tamsulosin group**	***Peganum*** ****** ***harmala*** ** group**	***P*** ** value**
	**Mean ± SD**	**Mean ± SD**	**Mean ± SD**
Before intervention	22.6 ± 3.8	21.5 ± 3.6	21.2 ± 4.4	0.347
After intervention	16.5 ± 3.7	12.0 ± 4.4	14.9 ± 5.3	0.001
*P* value	< 0.001	< 0.001	< 0.001	

Assessment of the survey scores for all groups before and after the intervention showed that they had significant difference (*P* < 0.05) and variables including urinary frequency and nocturia after the intervention, respectively with (*P* = 0.002) and (*P* = 0.001) in each three groups were significantly different. There was a significant difference among three groups in terms of intermittency symptoms (*P* = 0.002; Table 2).

**Table 2:** Comparison of IPSS before and after intervention in all three groups based on the survey questions

**Groups**	**Groups**			**P value**
	**Peganum harmala group (Mean±SD)**	**Peganum harmala and Tamsulosin group (Mean±SD)**	**Tamsulosin group** **(Mean±SD)**	
Incomplete discharge				
Before	2.68 ± 1.89	2.93 ± 1.70	2.68 ± 1.58	0.826
After	2 ± 1.70	1.86 ± 1.30	2.27 ± 1.46	0.568
*P* value before and after intervention	< 0.001	< 0.001	< 0.001	
Urinary frequency				
Before	3.65 ± 1	2.62 ± 1.42	3.51 ± 0.82	0.078
After	2.43 ± 1.01	1.68 ± 1.19	2.75 ± 0.91	0.002
P value before and after intervention	< 0.001	< 0.001	< 0.001	
Intermittency				
Before	2.28 ± 1.3	2 ± 1.33	2.96 ± 1.14	0.094
After	1.93 ± 1.13	1.10 ± 0.85	2.17 ± 0.88	0.001
*P* value before and after intervention	0.008	< 0.001	< 0.001	
Urgent need to urinate				
Before	2.87 ± 1.56	2.82 ± 1.25	2.89 ± 1.17	0.98
After	1.96±1.06	1.86 ± 0.63	2.41 ± 0.98	0.057
*P* value before and after intervention	< 0.001	< 0.001	< 0.001	
Weak flow of urine				
Before	3.56 ± 1.43	3.82 ± 1.03	4.10 ± 1.04	0.217
After	2.68 ± 1.37	2.34 ± 1.14	2.06 ± 1.06	0.141
*P* value before and after intervention	< 0.001	< 0.001	< 0.001	
Straining to urinate				
Before	2.46 ± 1.07	3.20 ± 1.26	2.58 ± 1.45	0.128
After	1.81 ± 1.17	1.86 ± 1.21	2.96 ± 1.05	0.871
*P* value before and after intervention	< 0.001	< 0.001	< 0.001	
Nocturia				
Before	3.68 ± 1.25	4.13 ± 0.91	3.86 ± 0.74	0.219
After	2.06 ± 1.13	1.34 ± 0.72	2.89 ± 0.72	0.001
*P* value before and after intervention	< 0.001	< 0.001	< 0.001	

## Discussion

This study examined the effect of *P. harmala* seed in benign prostatic hyperplasia.

In this study, combination of *P. harmala* with tamsulosin had more effect on alleviating mean of urinary symptoms than other groups, although this difference was significant in some cases. Besides, patients in *P. harmala* group compared to the third group had more average reduction in urinary symptoms after the intervention. Despite the fact that in some studies *P. harmala* has been known as a diuretic herb (16), in this study, it was suggested that it had a positive effect on variables such as frequent urination of patients.

On the other hand, the mechanism of action of benign prostatic hyperplasia is unknown, however, some studies considered the following factors including oxidative stress (17) inflammation (5) and vasoconstriction (18) in the occurrence of the disease. It was shown that ethanol extract of *P. harmala* seed can inhibit lipid peroxidation and improve the antioxidant property of *P. harmala* (19).

Bielli et al found that antioxidants are caused to widen arteries and have relaxant effect (20). In this study, according to relieve symptoms in patients using *P. harmala* and tamsulosin, it seems that the vasodilation effect of *P. harmala* triggered to improve symptoms. Bensalem et al have shown that anti-inflammatory properties of *P. harmala* are caused by the existence of β-carboline alkaloid that is due to inhibit myeloperoxidase and other factors involved in inflammation (21).

It is known that anti-inflammatory properties of this plant are caused by the inhibitory effect of some of inflammatory mediators such as prostaglandin E2 (PGE2) and tumor necrosis factor alpha (TNF-α) (22). In another study, it was found that alkaloid in *Peganum harmala* has relaxant effects on mice aorta (23).

Therefore, given to the afore-mentioned therapeutic properties, it is not far reached that *P. harmala* can be effective in reducing urinary symptoms in patients with benign prostatic hyperplasia. However, these therapeutic properties of *P. harmala* are more due to its alkaloid compounds such as harmalol, harmaline and harmine used in the treatment of cancer and benign prostatic hyperplasia. In addition, the other healing properties of this plant make it popular in the Iranian traditional medicine (14).

Other alkaloids found in *P. harmala* like vasicinone have anti-cell proliferation and can cause cytotoxicity in tumor cells in vitro (24). Of course, it should also be noted that β-carboline alkaloids in *P. harmala* can create toxicity at high doses (25), hence this herb should be administered in the effective therapeutic and non-toxic dose.

## Conclusion

In this study, most improvement of the symptoms was related to nocturia, urinary frequency and intermittency. According to the findings of this study, *P. harmala* can be used as a herbal drug for improving urinary symptoms of benign prostate enlargement considering its toxic dose.

## Limitations of the study

Lack of extraction and isolation of the main active compound of the plant and some defects in design of the study are the limitation of the study.

## Authors’ contribution

ZK; Study concept and design, acquisition of data, analysis and interpretation of data. SH; drafting of the manuscript. MS; critical revision of the manuscript for important intellectual content, administrative, technical, and material support.

## Conflicts of interest

The authors declared that there was no conflict of interest.

## Ethical considerations

Ethical issues (including plagiarism, data fabrication, double publication) have been completely observed by the authors.

## Funding /support

Shahrekord University of Medical Sciences supported this research financially (Grant # 1462, 2015).
